# Perianal Mucinous Adenocarcinoma Found Incidentally From Perianal Mass

**DOI:** 10.7759/cureus.48314

**Published:** 2023-11-05

**Authors:** Seyed Khalafi, Malini Riddle, Brittany Harper, Vid Fikfak

**Affiliations:** 1 Medicine, Paul L. Foster School of Medicine, Texas Tech University Health Sciences Center El Paso, El Paso, USA; 2 Surgery, Dartmouth-Hitchcock Medical Center, Lebanon, USA; 3 Surgery, Paul L. Foster School of Medicine, Texas Tech University Health Sciences Center El Paso, El Paso, USA

**Keywords:** perianal mucinous adenocarcinoma, anal carcinoma, incidental diagnosis, anal adenocarcinoma, anal fistula metastasis

## Abstract

Anal mucinous adenocarcinomas are very rare and usually arise from anal fistulas. We report a case of a 73-year-old man with a past medical history of hypertension admitted to our facility for evaluation of bleeding from a large, tender, left gluteal perianal mass. The patient reported the mass had been growing for over six years. On examination, an ulcerated, fungating large exophytic lesion was found extending from the anal verge laterally engulfing the left gluteus. The patient was anemic with low hemoglobin and hematocrit, as well as an elevated carcinoembryonic antigen level. A colonoscopy was performed during which an internal opening of a left-sided anal fistula was identified. The mass was biopsied and returned positive for a mucinous adenocarcinoma. Staging imaging including a computed tomography scan of the chest abdomen and pelvis did not show any metastatic disease. A magnetic resonance image of the pelvis revealed a locally invasive, heterogeneous tumor extending from the perianal soft tissue to the posterior wall of the anal canal and lower rectum. The patient was discussed at the interdisciplinary tumor board and completed five weeks of concurrent chemotherapy and radiation with 5-fluorouracil and a total of 28 fractions of radiation. He then underwent abdominoperineal resection with a vertical rectus abdominis myocutaneous flap. The patient was placed in the surgical intensive care unit and subsequently discharged in stable condition on postoperative day 14. This case highlights the presentation, diagnosis, and management of anal mucinous adenocarcinoma.

## Introduction

Mucinous adenocarcinoma is defined histologically as mucinous lakes with clusters of cancer cells [1]. These types of cancers can be found in any part of the gastrointestinal tract and make up 2-3% of all gastrointestinal tumors [2]. They may also be found extraintestinal, including the breast, thyroid, or skin. Of these locations, the perianal region is considered the rarest, making up 3-11% of all perianal cancers [1,2]. Perianal mucinous adenocarcinoma (PMA) is a subtype of colorectal cancer that arises from the stratified columnar epithelium lining of the anal glands and accounts for 6.9% of all anal cancers. This subtype is also considered more aggressive than epidermoid cancers [3,4].

The pathogenesis of this cancer is debated but may be due to chronic inflammation, with some case reports linking chronic anal fistulation as an etiology [2]. Other pathologies that are supposedly associated include perianal abscesses [5] or perianal Crohn’s disease [6]. No associated risk with smoking, alcohol use, obesity, or increased red meat intake has been identified [2]. In addition to inflammation, a genetic component may also play a role. One suggested gene that may contribute to pathogenesis is overexpression of the mucin 2, oligomeric mucus gel-forming (MUC2) protein [7]. Cells in PMA have also been observed to have high rates of microsatellite instability, similar to Lynch syndrome, and mutations of the Ras/mitogen-activated protein kinase (MAPK) pathway [8]. These etiologies may explain the disease course going undetected for years as clinicians focus on chronic inflammatory diagnoses rather than cancer [9]. We report a case of a 73-year-old male with anal mucinous carcinoma arising from an anal fistula.

## Case presentation

**Figure 1:**
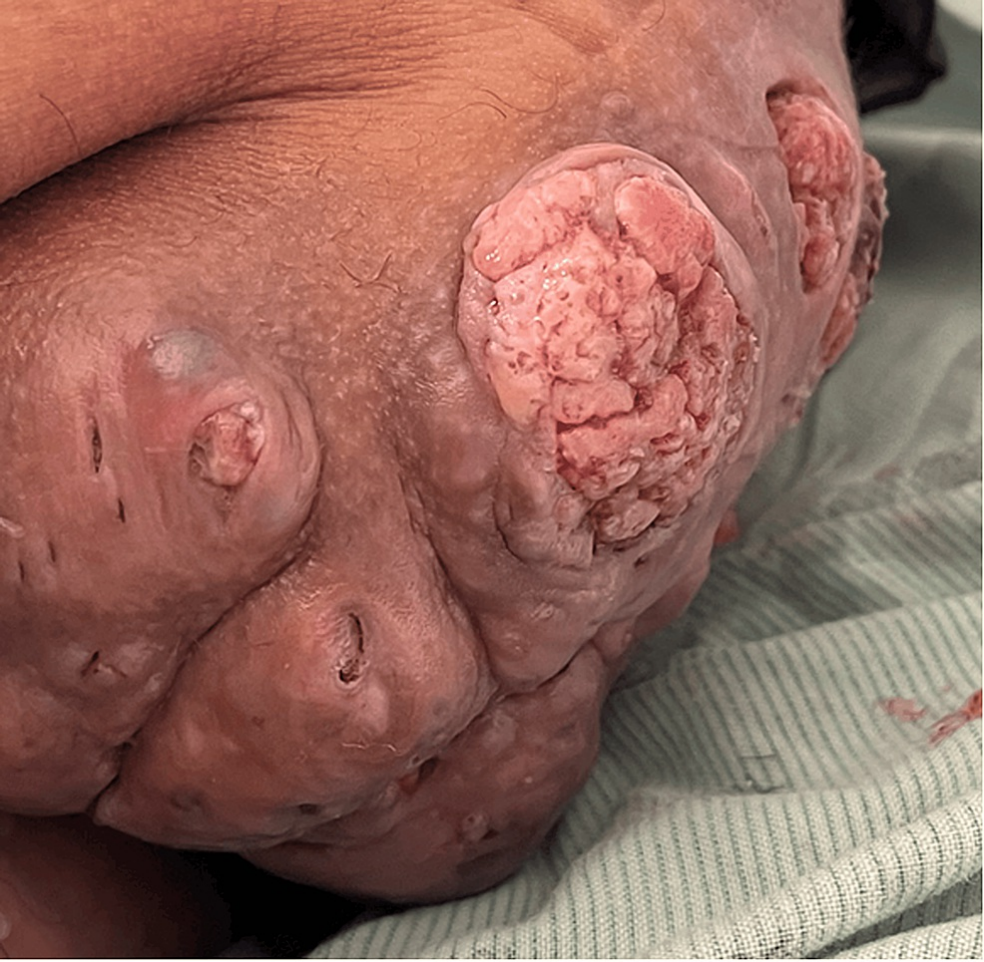
Large, ulcerating, and fungating left gluteal exophytic lesion extending from the perirectal region.

A 73-year-old male with a past medical history of hypertension presented to the hospital with a six-year history of a painful, bloody left gluteal perirectal mass. It had increased in size in the past four to six months, during which time he had also noted fluctuating weight with a recent unexplained weight loss of 6 kg. He denied any fatigue, constipation, bloody stools, or melena. The patient also stated that he had never had a colonoscopy and had no family history of cancer. Reportedly, the mass had been biopsied previously in Mexico with benign results. The patient was hypothermic, with a temperature of 35.0 degrees Celsius, bradycardic (53 beats/min), and hypertensive (181/81 mmHg). On physical examination, a large exophytic mass extending from the perirectal region to the left gluteus was noted. The mass was large, ulcerated, and fungating and was approximately 15 cm in greatest diameter (Figure 1).

On digital rectal exam, the anal canal was patent and the mucosa smooth; however, despite feeling the mass, we could not ascertain if it was originating from the anal canal or the lower rectum. The rest of the physical exam was within normal limits.

Admission lab results showed a normal white blood cell count and low hemoglobin and hematocrit of 9.5 g/dL and 32.3%, respectively. Serum sodium and potassium levels were also within normal limits, in addition to creatinine, blood urea nitrogen, and liver enzyme levels. Subsequent lab results did report a high erythrocyte sedimentation rate (ESR) of 80 mm/hr and carcinoembryonic antigen (CEA) of 73.4 ng/mL.

**Figure 2:**
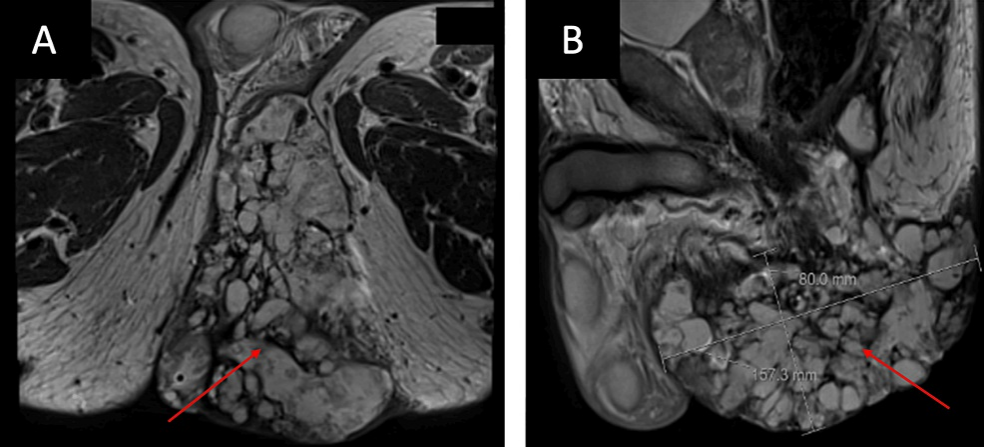
An axial (A) and sagittal (B) view of the pelvic magnetic resonance imaging showing a large T2 hyperintense, locally invasive, heterogeneous tumor (red arrow) infiltrating the posterior wall of the anal canal and lateral walls of the lower rectum.

A pelvic MRI with and without contrast revealed a large T2 hyperintense, locally invasive, heterogeneous tumor measuring 15.7 x 10.5 x 8 cm. The mass was centered in the perianal soft tissue involving the left gluteal fold to the overlying skin. The tumor extended through the internal and external sphincters to the posterior wall of the anal canal, abutting the puborectalis and infiltrating the posterior wall of the anal canal and lateral walls of the lower rectum (Figure 2).

Punch biopsy subsequently resulted as invasive, well-differentiated mucinous adenocarcinoma without involvement of epidermis, most compatible with colorectal adenocarcinoma. CT of the chest/abdomen/pelvis showed no evidence of metastatic disease apart from the rectal mass. Due to the patient’s extensive progression of his tumor, it was difficult to clinically stage the patient’s PMA. The patient was discharged and advised to follow up outpatient for further workup.

**Figure 3:**
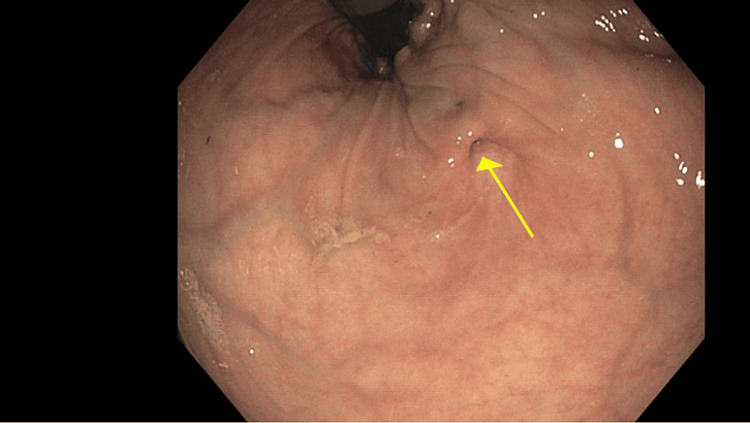
Fistula (yellow arrow) noted in the rectum 2-3 cm proximal to the anus on colonoscopy.

On outpatient follow-up, the patient underwent a colonoscopy, revealing no involvement of the anal canal. A fistula was also found in the rectum, located 2-3 cm proximal to the anus (Figure 3).

**Figure 4:**
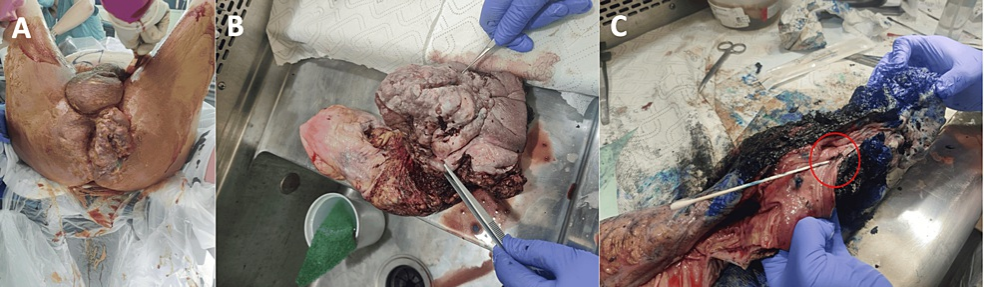
Patient was prepped (A) and underwent robot-assisted APR with VRAM flap, perineal resection of additional tumor involving the skin, subcutaneous tissue, fascia, and muscle with total defect measuring 400 cm² (B), coccygectomy, and end colostomy creation. Fistula was noted with cotton swab (C) (red circle). APR: abdominoperineal resection; VRAM: vertical rectus abdominis myocutaneous

The patient completed five weeks of neoadjuvant concurrent chemoradiation with 5-fluorouracil (5-FU) infusion and 28 fractions of radiation, resulting in a moderate reduction in tumor size based on imaging obtained after administration of therapy. He then underwent a robot-assisted abdominoperineal resection (APR) followed by an open vertical rectus abdominis myocutaneous (VRAM) flap. During this, the entire tumor along with the coccyx that was involved was removed and an end colostomy was created (Figure 4).

The patient did well after surgery; however, he did develop a postoperative ileus which was treated with gastric decompression via nasogastric tube placement. He then recovered and was discharged home in stable condition on postoperative day 14. The final pathology was significant for moderately differentiated adenocarcinoma (50%-95% gland formation) invading the muscularis propria and extending into the subserosal soft tissue. Twelve lymph nodes were identified and all were negative for metastasis. All resection margins were negative with the closest margin 1 cm away from the tumor. The final staging was ypT3 and ypN0. The patient was categorized as having a fistula-in-ano subtype of PMA with malignant degeneration.

## Discussion

PMA is a rare tumor that may go undetected for a long period of time or mimicking other common chronic inflammatory conditions. The growth of the tumor is indolent and is usually contained within the perianal tissue [2]. Classic signs and symptoms of PMA include perianal pain, itching, mucinous discharge/abscess, and a palpable mass in the perianal region [1]. PMA also uncommonly presents with anal bleeding due to rare mucosal involvement of the rectum or anus [10]. As for our patient, he presented with a painful perianal mass with bloody discharge, but no itchiness was reported. For six years, our patient’s only condition was a perirectal mass with no detection, which eventually worsened, resulting in an increase in size, bloody discharge, and weight loss.

The formation of new ulcero-proliferative growth, such as persistent fistula in ano, is a clinical sign that usually raises suspicion and need for further workup [2]. Anal fistulas account for only 6.9% of all cancers in the anal canal [11]. Mucinous adenocarcinoma should be suspected in perianal fistulas with atypical and recurrent clinical features. More than 10 years after the onset of anal fistula, the risk of developing PMA increases [5]. A literature review of cases from 2018 showed that out of 34 cases of PMA, 31 cases were found to have a fistula on diagnosis (Table 1).

**Table 1:** Association of mucinous adenocarcinoma with perianal fistula in relevant studies from 2018 to 2023 NR: not reported; M: male; F: female, APR: abdominoperineal resection, VRAM: vertical rectus abdominis flap; TRAM: transverse rectus abdominis flap; 5-FU: 5-fluorouracil

Author	Number of cases	Age	Sex	Fistula	Lymph node involvement	Metastasis	Treatment	Chemo/radiotherapy
Díaz-Vico et al. [1]	3	66, 71, 62	M, M, M	Yes, Yes, Yes	No, No, No	No, No, No	ischioanal APR and a VRAM flap, ischioanal APR and VRAM flap, ischioanal APR and VRAM flap	neoadjuvant chemoradiotherapy with capecitabine, neoadjuvant chemoradiotherapy, postoperative capecitabine
Cenac et al. [4]	1	44	F	Yes	Yes	NR	elliptical incision	NR
Koizumi et al. [5]	1	68	F	Yes	Yes	No	APR	adjuvant chemotherapy with 5-FU, leucovorin, oxaliplatin, and irinotecan with bevacizumab
de Souza et al. [6]	1	71	M	Yes	Yes	No	APR with partial prostatectomy	adjuvant treatment with capecitabine
Alvarez-Laso et al. [11]	4	80, 40, 66, 71	M, M, M, M	Yes, Yes, Yes, Yes	Yes, No, No, Yes	No, No, No, No	APR, APR, APR, APR	adjuvant chemoradiotherapy with 5-FU, NR, neoadjuvant chemoradiotherapy with 5-FU, neoadjuvant chemoradiotherapy with 5-FU
AlSalim et al. [12]	1	43	M	Yes	No	No	APR	neoadjuvant chemoradiotherapy with capecitabine
Feo et al. [13]	1	80	M	Yes	No	No	multiple endoscopic polypectomies and APR	adjuvant radiotherapy with capecitabine
Prasad et al. [14]	1	34	F	Yes	No	No	APR	neoadjuvant chemoradiation with oxaliplatin, capecitabine and dexamethasone
Kim et al. [15]	1	80	F	No	NR	NR	full- thickness 20 cm 2 wide local excision with V-Y advancement flap and an inferior- based rotation	NR
Azmi et al. [16]	1	66	M	Yes	No	No	APR with VRAM flap	None
Jee et al. [17]	1	29	M	Yes	Yes	No	APR	neoadjuvant and adjuvant chemoradiation
Matos-Pires et al. [18]	1	77	F	No	NR	Yes	NR	NR
Toyonaga et al. [19]	3	54, 48, 59	M, M, M	Yes, Yes, Yes	No, No, No	No, No, No	APR, APR, APR	NR, NR, NR
Chrysikos et al. [20]	1	65	M	Yes	No	No	APR	None
Au et al. [21]	1	66	M	Yes	No	No	APR with TRAM flap and uretherectomy, cystoprostatectomy with ileal conduit formation	neoadjuvant chemoradiotherapy with capecitabine
Komornik et al. [22]	1	72	M	Yes	Yes	No	APR	neoadjuvant chemo-radiotherapy with capecitabine and adjuvant chemoradiotherapy with oxaliplatin/leucovorin/fluorouracil
An et al. [23]	2	79, 42	M, M	Yes, Yes	No, No	No, No	APR, APR with V-Y advancement flap	NR, adjuvant chemotherapy with 5-FU
Deng et al. [24]	1	68	M	No	NR	No	APR	NR
Zhu et al. [25]	8	NR	NR	8 cases with	5 cases with	NR	NR	NR

The cause of the malignant transformation from fistulas is not fully understood, but it may be due to the dysplastic degeneration of cells [11].

Another reason the tumor may go unnoticed is due to the failure to obtain biopsy evidence. Establishing early clinical suspicion is key to begin obtaining biopsies, which are difficult to obtain with this type of cancer. Cancer cells are usually located deep in the tissue, and for this reason, many biopsies fail to capture the picture of the cancer. The case report by Okada et al. showed no significant clinical evidence of PMA from four biopsies, resulting in a delay in diagnosis [10]. Similarly, our patient had an initial biopsy done that showed no abnormalities. Due to the tumor being difficult to biopsy, it may be beneficial to generously perform biopsies to prevent misdiagnosis [5]. While biopsy is essential for diagnosis, there are multiple other studies that may help with diagnosis. Endoscopic ultrasound (EUS), CT, and MRI are the gold standard for diagnosing PMA, with MRI being the most sensitive in portraying the mucinous structures [1]. Positron emission tomography (PET) scans have not been seen to be significantly helpful in diagnosing PMA due to the mucin 2-[fluorine-18]-fluoro-2-deoxy-D-glucose (FDG) uptake and false negative results [26]. An MRI was conducted on our patient which was able to note the extent of the tumor as well as the content. On noticing the extent of the tumor, a biopsy was conducted, which confirmed the diagnosis. While metastasis to inguinal lymph nodes may occur, distal metastasis is uncommon [1].

Neoadjuvant chemotherapy has emerged as having been shown to better control the disease [27]. A randomized trial by Bujko et al. showed significant benefit in local control and sphincter preservation of adding 5-FU and leucovorin chemotherapy to rectal cancers preoperatively without significant difference in disease-free or overall survival and stoma placement [28]. Post-surgical adjuvant has been considered a mainstay of treatment for rectal adenocarcinoma along with neoadjuvant chemoradiation therapy [2]. Our patient was not given adjuvant therapy due to surgical resection showing negative margins and no lymph node metastasis. 

According to Ilbawi et al., PMA can be classified as duct, ectopic perianal/ischiorectal, or associated with fistula in ano [29,30]. The anal duct subtype is considered aggressive and is suggested to be treated with APR and adjuvant chemoradiotherapy. The ectopic subtype is known to have a high local recurrence rate and should also be considered for APR with wide local excision and negative margins. The fistula in ano subtypes can be further subclassified as seeding from colorectal adenocarcinoma or malignant degeneration. The seeding subtype can be considered for resection of the primary colorectal or anal tumor and fistula in ano satellite site, while the degenerative subtype warrants APR with wide local excision. The use of sentinel lymph node biopsy is also not recommended for all subtypes. While there is no general consensus on treatment for the mentioned subtypes, the current standard of care is APR [30]. Due to PMA’s indolent course, prognostics of the tumor have not been well established, with one case report stating an almost 100% mortality in one year [31].

In our case, even despite the large size of the tumor, combining a robot-assisted low anterior resection with an extensive perineal resection allowed us to achieve an excellent oncologic outcome by obtaining negative margins. This approach has the added benefit of allowing two surgeons to work together, one performing the abdominal resection and the other the perineal resection until the two resections meet and the specimen can be removed en block.

Once the procedure is complete there have been benefits shown for using a VRAM flap versus a primary closure. VRAM flaps have been used commonly to help bring well-vascularized tissue closer together and to minimize the gap between tissues from large resections. It is also helpful in patients who undergo neoadjuvant therapy with damage to the skin and possible postoperative complications, such as wound dehiscence, abscesses, and perineal sinuses. A cohort study by Kent et al. reported that patients who had VRAM flap reconstruction were less likely to have parastomal and perineal hernia rates than patients who underwent laparoscopic or open APR with primary closure [31]. Our patient underwent a VRAM flap placement after APR postoperatively and was stably discharged from the surgical intensive care unit with no postoperative complications.

## Conclusions

In this case report, we present a rare type of colorectal cancer with a unique management to prevent pre- and postoperative complications. Our patient did not present with typical signs of cancer and had inconclusive tests done outside the country which delayed his diagnosis and treatment until he presented to our hospital. Our case also shows that even large tumors of the anal canal that involve extensive perianal area may be amenable to surgical resection where negative margins can be achieved. With PMA being a very rare, aggressive, and poorly diagnosed tumor, we hope to promote the improvement of diagnosis and management of this disease.
